# Spectral Reconstruction Using an Iteratively Reweighted Regulated Model from Two Illumination Camera Responses

**DOI:** 10.3390/s21237911

**Published:** 2021-11-27

**Authors:** Zhen Liu, Kaida Xiao, Michael R. Pointer, Qiang Liu, Changjun Li, Ruili He, Xuejun Xie

**Affiliations:** 1 School of Statistics, Qufu Normal University, Qufu 273165, China; zhen@whu.edu.cn (Z.L.); liuqiang@whu.edu.cn (Q.L.); xuejunxie@126.com (X.X.); 2 School of Electronics and Information Engineering, University of Science and Technology Liaoning, Anshan 114051, China; cjliustl@sina.com; 3 School of Design, University of Leeds, Leeds LS2 9JT, UK; mrpointer@btinternet.com (M.R.P.); sdrh@leeds.ac.uk (R.H.)

**Keywords:** spectral reconstruction, two illuminations, iteratively reweighted regulated model, feature selection, RGB images

## Abstract

An improved spectral reflectance estimation method was developed to transform captured RGB images to spectral reflectance. The novelty of our method is an iteratively reweighted regulated model that combines polynomial expansion signals, which was developed for spectral reflectance estimation, and a cross-polarized imaging system, which is used to eliminate glare and specular highlights. Two RGB images are captured under two illumination conditions. The method was tested using ColorChecker charts. The results demonstrate that the proposed method could make a significant improvement of the accuracy in both spectral and colorimetric: it can achieve 23.8% improved accuracy in mean CIEDE2000 color difference, while it achieves 24.6% improved accuracy in RMS error compared with classic regularized least squares (RLS) method. The proposed method is sufficiently accurate in predicting the spectral properties and their performance within an acceptable range, i.e., typical customer tolerance of less than 3 DE units in the graphic arts industry.

## 1. Introduction

One of the ultimate goals of spectral estimation from a camera image is to predict the spectral reflectance data that represent the physical properties of a device-dependent camera signal. Spectral reflectance is a major area of interest in many fields, including biometric identification [1,2], art archiving [3], cosmetics [4,5], agriculture [6], and high-fidelity color reproduction [7]. Thus, the technique of spectral estimation from camera images has gained importance.

Generally, it is acknowledged that spectral reflectance estimation from a three-channel RGB camera has a relatively lower estimation accuracy compared with a multispectral imaging system that contains more signal channels with different filtration mechanisms. Conventionally, a multispectral imaging system is constructed using a camera with bandpass filtration systems, such as narrowband filters, liquid crystal tunable filters (LCTF) [7,8,9,10,11], programmable illumination imaging system [12,13,14,15,16,17], or broadband filters with trichromatic cameras [18,19,20,21,22,23]. It is worth mentioning that, with dramatically developing technology for both digital cameras and LED lighting, a multispectral imaging system with consumer-grade cameras and broadband lighting can be developed with much lower cost and easier operation processing, although the spectral reflectance estimation algorithm needs to be developed and verified.

The current literature on spectral reflectance estimation is extensive and focuses particularly on accurate algorithms. These algorithms can be classified into three groups: model-based methods, interpolation methods, and learning-based methods [20]. Recently, learning-based reconstruction has developed dramatically. It can use low-cost but high-resolution cameras, and it is convenient in practical applications because it is not necessary to characterize the spectral sensitivity function of the imaging system, which has a low requirement in wavelength resolution similar to a spectrophotometer. The performance, however, is greatly affected by the choice of the training set used as part of the characterization process. In recent years, there has been an increased amount of literature on learning-based algorithms, for example, the pseudoinverse method, Wiener estimation [23,24], principal component analysis [25,26,27], and polynomial-based regression models [18,28,29]. For example, Berns et al. proposed an image-based spectral reflectance estimation method using matrix R based on the Wyszecki hypothesis by combining a trichromatic camera and absorption filters [18,19]. Hardeberg et al. proposed a method using a principal eigenvector technique and pointed out that any spectral reflectance can be expressed as a linear combination of basic functions and a scalar vector and evaluated illuminant estimation models from color to multispectral imaging [7,10]. Li and Cao proposed two reconstruction methods, based on local linear regression, which achieve reasonable reconstruction accuracy [30,31]. Shen et al. reported that the partial least squares regression (PLS) method could also be adopted in constructing a regression model based on the correlation between response value and spectral reflectance [32]. All these studies claimed to have achieved good results using different metrics.

Overall, most studies use a mathematical algorithm for spectral estimation. However, the impact of overfitting, which would lead to poor accuracy performance, has not been sufficiently investigated. Overfitting is usually caused by the polynomial degree of camera signal expansion. When using higher degree polynomials, the irrelevant detail and noise in the training dataset are picked up and learned as concepts, and the error for the test set starts to rise as the model’s ability to generalize decreases, i.e., when a model works well for the training set data but performs badly on the test set. Several approaches could be used to reduce this problem, such as approaches based on reducing the feature numbers or imposing penalties by putting weights on the features, such as the regularization method [33,34,35,36,37,38,39,40]. For example, Shen et al. proposed a nonlinear regression method based on a polynomial model for spectral estimation, with consideration being given to the potential overfitting problem in the polynomial-based regression model [14,33]. Graham et al. demonstrated that the root-polynomial regression model could provide leading performance in both spectral recovery and color reproduction. Harifi et al. initially applied the principal component analysis embedded regression method to recover the spectral reflectance, and a third-order polynomial system was found to be best for the calculation of the transformation matrix [28]. Therefore, a method that reduces the problem of overfitting as well as characterizes the nonlinearity of the spectral reflectance estimation of multispectral imaging systems should be promising.

The main aim of this study is to develop a multispectral imaging system using an RGB camera and two commonly used illuminants. We specifically focused on the development of a more accurate spectral estimation method from raw camera responses using the iteratively reweighted regularization regression model proposed in this study. To solve the overfitting problem, we develop a feature selection process that uses neighborhood component analysis. The superior performance of this proposed method is evaluated and compared with existing methods by using both a semiglossy ColorChecker SG (CCSG140) chart and a matte ColorChecker DC chart (CCDC240). The overall performance of both the proposed and the traditional methods is compared in terms of both spectral and colorimetric accuracy.

## 2. Multispectral Imaging Model

In this section, we show the details of the proposed method. Two raw images are captured under two different color temperature lighting conditions to recover the spectral reflectance of a scene.

### 2.1. Regularization Model

The camera response is proportional to the intensity of the captured light; thus, the camera response can be expressed as a linear combination of the camera sensitivity functions, the illumination spectral power distribution, and the spectral reflectance of the objects. As has been shown in previous work [5,6,7,8,9,10], the camera sensor response vector *c* can be formulated as spectral reflectance:

(1) c=rLS+ε

where *S* is a 31 × 3 matrix that represents the spectral sensitivities of the sensors (assuming we have 31 bands from 400 nm to 700 nm at 10 nm intervals); *L* is a 31 × 31 diagonal matrix that represents the spectral power distribution of the illuminant; *r* is a 1 × 31 discrete spectral vector of the object uniformly sampled over the visible wavelength range, typically from 400 nm to 700 nm at 10 nm intervals; *ε* is a 1 × 3 vector of the additive system errors; and *c* is a 1 × 3 vector that represents the camera sensor response.

The spectral reflectance can be estimated using prior knowledge of a training set of measured color patches and camera responses. When the error can be ignored, the above equation can be transformed as a scalar product in matrix notation as:

(2) R=CQ

where *R* is an *n* *×* 31 matrix of the spectral dataset in which each row represents the spectral reflectance of *n* samples. *C* is an *n ×* 3 matrix that represents the camera response, and each row consists of one or two sets (two three-channel images taken under two different lighting conditions) of camera responses for a sample. *Q* is a 3 × 31 matrix that represents the transform between the camera response and the spectral reflectance.

As described in the literature, most spectral estimation processes perform spectral estimation based on regression models, including linear, second-, third-, and fourth-order polynomial expansion models. In general, the accuracy of the spectral estimation depends not only on the training set but also on the number of signal features: the greater the number of features, the better the linearity of the imaging system. To improve the spectral estimation accuracy, a polynomial transform was used to extend the response values instead of increasing the number of imaging channels. Taking a third-order polynomial model as an example, the six-channel camera response (a row of *C*) can be expanded to 84 terms:

(3) $$D=\left[1,R_{1},G_{1},B_{1},R_{2},G_{2},B_{2},R_{1}^{2},R_{1}G_{1},\cdots G_{2}B_{2},B_{2}^{2},\cdots ,R_{1}^{3},R_{1}^{2}G_{1},R_{1}^{2}B_{1}\cdots B_{2}^{3}\right]$$

Using *D* to denote the matrix expanded from *C*, models can be built to map the polynomial camera response features to the spectral reflectance as:

(4) R=DΘ

where *Θ* is the 84 × 31 transform matrix searched for by the least-squares method. This could address some of the problems by imposing a penalty on the size of the coefficients. The *L*2-norm of vector *κ* can be added to the loss expression to give the preferred solutions with smaller norms. The objective functions $J_{1}(\Theta )$ are as follows:

(5) $$J_{1}(\Theta )=\arg\min\left({\left\|D\Theta -R\right\|}_{2}^{2}+\kappa {\left\|\Theta \right\|}_{2}^{2}\right)$$

here, *κ* is a regularization parameter to be empirically selected. The purpose of this regularization setting is to stabilize the regression output, which prevents a large change in the result when small perturbations in the input camera response occur. The gradient of the objective functions in the least-square sense becomes:

(6) $$\frac{\partial J_{1}(\Theta )}{\partial \Theta }=\frac{\partial }{\partial \Theta }({\left(D\Theta -R\right)}^{H}(D\Theta -R)+\kappa \Theta^{H}\Theta )=D^{H}D\Theta -D^{H}R+\kappa \Theta$$

Let the gradient manually be zero, that is, $\frac{\partial J_{1}(\Theta )}{\partial \Theta }=0$

(7) $$\Theta ={\left(D^{H}D+\kappa I\right)}^{-1}D^{H}R$$

where *I* is the identity matrix. Small, positive values of *κ* reduce the variance of the estimates. While biased, the reduced variance of ridge estimates often results in a smaller mean squared error when compared to the ordinary least-squares estimates, and $\delta_{\min}$ is the smallest positive singular value. Referring to the singular value decomposition of the expanded camera signal response matrix, $D=U\Sigma V^{H}$, $D^{H}D=V\Sigma^{H}\Sigma V^{H}$, the above equation can be transformed:

(8) $$\Theta =V{\left(\Sigma^{H}\Sigma +\delta_{\min}^{2}I\right)}^{-1}\Sigma^{H}U^{H}R$$

### 2.2. Iteratively Reweighted Regularization Model

The conventional method is optimum when the noise is ignored; however, it provides a poor estimation and is unreliable when outliers are present in the training data. Residual analysis is required to address these problems and downweight the influence of outliers. Here, we proposed a method by assigning a weight to each training sample to downweight the influence of outliers iteratively. In the first iteration, each training sample is assigned an equal weight, and the model coefficients are estimated using the regularization model from Equation (8). At subsequent iterations, weights are recomputed so that the points farther from the model predictions in the previous iteration are given lower weight until the values of the coefficient estimates converge within a specified tolerance. The modified objective function $J_{2}(\Theta )$ minimized by the M-estimator is as follows:

(9) $$J_{2}(\Theta )=\arg\min\left[\omega \left({\left\|D\Theta -R\right\|}_{2}^{2}+\kappa {\left\|\Theta \right\|}_{2}^{2}\right)\right]$$

where *ω* is a function of weighted residuals called fair estimators defined as follows. The M-estimator needs to be found; it is a way of mitigating the influence of outliers in an otherwise normally distributed data set.

(10) $$w=\frac{1}{1+\left|u\right|}$$

The value *u* in the weight functions is

(11) $$u=\frac{resid}{tune\times s\times \sqrt{1-h}}$$

where *resid* is the vector of residuals from the previous iteration, the *tune* is the tuning constant with 1.4 as the default, and *s* is an estimate of the standard deviation of the error term given by

(12) $$s=\frac{median\left[abs\left(resid/\sqrt{1-h}\right)\right]}{0.6745}$$

where *h* is the vector of the leverage values from a least-squares fit, which is calculated from a QR decomposition, and $h=sum(Q^{2})$. Assuming the signal matrix is of full rank and the test sample errors are independent and identically distributed with variance, the gradient for the weighted residual in the least-square sense becomes

(13) $$\begin{array}{l} \frac{\partial J_{2}(\Theta )}{\partial \Theta }=\frac{\partial }{\partial \Theta }\left[{\left(wD\Theta -wR\right)}^{H}(wD\Theta -wR)+\kappa w\Theta^{H}\Theta \right]\\ \begin{array}{ccc} & & = \end{array}\frac{\partial }{\partial \Theta }\left[\Theta^{T}D^{T}w^{T}wD\Theta -2R^{T}w^{T}wD\Theta +R^{T}w^{T}wR+\kappa w\Theta^{H}\Theta \right]\\ \begin{array}{ccc} & & = \end{array}D^{T}w^{T}wC\Theta -D^{T}w^{T}wR+\kappa \Theta \end{array}$$

Let the gradient of the above equation be zero. The schemes for finding the solution are as follows:

(14) $$\Theta ={\left[D^{T}w^{T}wD+\kappa I\right]}^{-1}D^{T}w^{T}wR$$

Solving this estimation equation is equivalent to a weighted least-squares problem: the weight depends upon the residuals, and the residual depends upon the estimated coefficient, so an iterative solution is therefore required.

(a) The initial estimates of spectral reflectance *R*^0^ are calculated in Equation (8).

(b) At each iteration *t*, residuals *resid*^(*t*−1)^ and associated weights *w_i_*^(*t*−1)^ are calculated from the previous iteration.

(c) The new weighted least-squares estimates are solved with Equation (10)

(15) $$\Theta^{t}={\left[D^{T}w^{\left(t-1\right)}{}^{T}w^{\left(t-1\right)}D+\kappa I\right]}^{-1}D^{T}w^{\left(t-1\right)}{}^{T}w^{\left(t-1\right)}R$$

(d) Steps (b) and (c) are repeated until the estimated coefficients converge.

### 2.3. Feature Selection

It should be noted that when the number of polynomial expansion signals is large, its components are correlated, and the columns of the signal matrix have an approximately linear dependence. The estimation is extremely sensitive to random noise in the camera response, producing a large variance, and the situation of multicollinearity is an issue. This will degrade the prediction performance and the stability of spectral estimation precision [29]. The objective of a feature selection search for a subset of extended polynomial camera responses is to optimally model the camera responses and the spectral reflectance. The subset is subject to constraints such as the required or excluded features and the size of the subset. The performance of the spectral estimation transform matrix can be improved using the neighborhood component analysis feature selection [38,39,40,41,42]. Consider a spectral estimation training set *S* containing *n* color patches:

(16) $$S=\left\{(c_{i},r_{i}),\;i=1,2,3\cdots n\right\}$$

where *c_i_* is the polynomial expansion of the camera signals from the *i*th patch, and *r_i_* is the corresponding spectral reflectance. A randomized regression model can be built as follows:

(a) A patch *Ref*(*c*) is randomly selected from *S* as the ‘reference point’ for camera response.

(b) The response value at *c* is set equal to the response value of reference point *Ref*(*C*).

The probability, *P*(*Ref* (*x*) *= c_j_|S*), that point *c_j_* is picked from *S* as the reference for *c* is

(17) $$P\left(Ref(c)=c_{j}|S\right)=\frac{\upsilon (d_{w}(c,c_{j}))}{\sum_{j=1}^{n}\upsilon (d_{w}(c,c_{j}))}$$

where *d_w_* is the distance function, and *ν* is the kernel function that assumes large values when *d_w_* is small. Now consider the leave-one-out application of this randomized regression model, that is, predicting the response for $c_{i}$ using the data in $S^{-i}$, and the training set *S* excluding the point $\left(c_{i},r_{i}\right)$. The probability that point *c_j_* is picked as the reference point for $c_{i}$ is given by:

(18) $$p_{{}_{ij}}=P(ref(c_{i})=c_{j}|S^{-i})=\frac{\upsilon (d_{w}(c_{i,}c_{j}))}{\sum_{j=1,j\neq i}^{n}\upsilon (d_{w}(c_{i,}c_{j}))}$$

Let ${\tilde{r}}_{i}$ be the response value the randomized regression model predicts and *r_i_* be the actual spectral response for *c_i_*. Let *l* be a loss function that measures the disagreement between *r_i_* and ${\tilde{r}}_{i}$. Then, the average value of the loss function *l*(*r_i,_* ${\tilde{r}}_{i}$) is given by:

(19) $$l_{i}=E(l(r_{i},{\tilde{r}}_{i})|S^{-i})=\sum_{j=1,j\neq i}^{n}p_{ij}l(r_{i},{\tilde{r}}_{i})$$

After adding the regularization term *λ*, the weight vector *w_f_* can be expressed as the following minimization regression error:

(20) $$w_{f}=\underset{w_{f}}{\arg\min}\{f(w_{f})\}=\underset{w_{f}}{\arg\min}\{\frac{1}{n}\sum_{i=1}^{n}l_{i}+\lambda \sum_{r=1}^{p}w_{r}^{2}\}$$

where *w_r_* is weight vector for *r*th feature item, *n* is the number of observations, and *p* is the number of predictor variables.

## 3. Experiment and Result

In this section, the proposed method is implemented and compared with the currently existing methods; meanwhile, the feature selection that will influence the estimation accuracy of the proposed method is also investigated and discussed.

### 3.1. Camera Setup

**Figure 1:**
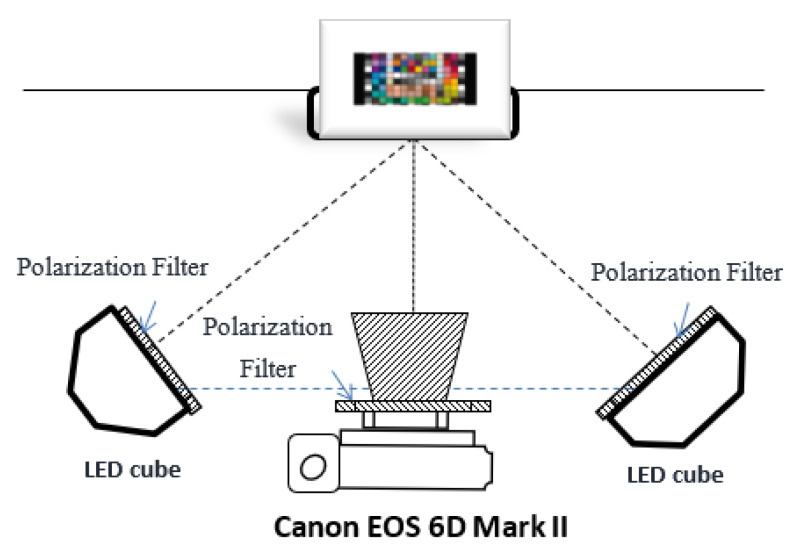
The system setting of the image acquisition.

To verify the proposed approach, comparative experiments were conducted. The multispectral imaging system we developed includes a commercial trichromatic camera (Canon EOS 6D Mark II) with 16-bit digitization and two spectrally tunable THOUSLITE LED Cubes mounted with translucent diffuse reflectors. To illuminate glare and specular highlights, a linear polarizer was placed in the illumination plane of each LED Cube with the polarizing axes orientated in the same direction, and another linear polarizing filter was placed on the lens of the camera. As shown in Figure 1, the imaging plane of the digital camera was set to be approximately parallel to the sample placement plane, and the two LED Cubes were placed at an angle of approximately 45° to the color samples.

**Figure 2:**
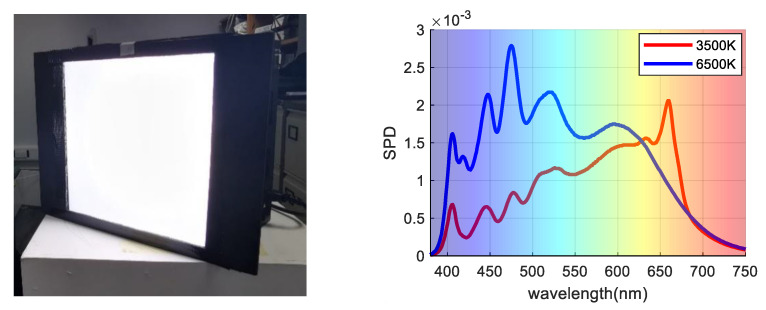
(**Left**) LED Cube; (**Right**) Two SPD curves under 3500 K and 6500 K illumination conditions.

During image capture, each cube is used to simulate D65 (CCT approximately 6500 K) and incandescent light (CCT approximately 3500 K), These two illuminations are most commonly used, and the shape of SPD curves are quite different and weak in correlation. The spatial nonuniformity of the light field was corrected using exposure to a white card. The relative spectral power distribution of the two light sources was measured using a diffused white sample and is illustrated in Figure 2. Note that the SPD curves are similar in some wavelength bands because these lights are fitted from 15 narrow channels over the visible wavelength range.

The parameter settings for the camera were fixed during the image capture, with the aperture size set to f5.6, the shutter speed at 1/8 s, and the ISO speed set to 640. Canon EOS Utility software was used to control the camera for the image capture. The original raw responses were recorded by the camera and used to predict spectral reflectance. The dark current noise was recorded with the camera lens cap closed and was subsequently subtracted from the captured digital images.

**Figure 3:**
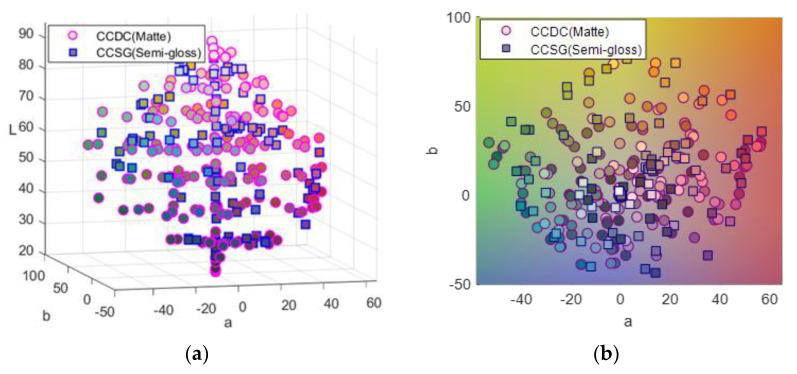
The color distribution of matte charts (circle marker) and semigloss charts (square maker). (**a**) Comparison of color distribution in the CIELAB color space. (**b**) The chromaticity coordinates of samples in a* − b* plane.

An X-Rite ColorChecker semigloss chart (CCSG, 140 patches) and a Gretag–Macbeth ColorChecker DC matte chart (CCDC, 240 patches including 232 mattes, and 8 glossy patches) were used as color targets and captured by the proposed multispectral imaging system. The spectral reflectance of all the patches was measured by a Konica Minolta CM2600D portable sphere Spectrophotometer from 400 nm to 700 nm (under SCI measurement based on diffuse: 8 geometry) at intervals of 10 nm over the wavelength range. Measuring Specular Component Included (SCI) would capture true color data from the sample and negate the effect of surface appearance to measure only color. It makes little or no difference if the patches are mirror-like or matte in appearance. Figure 3 shows the color distribution of both charts plotted in the CIELAB color space. The CIELAB values were calculated using the CIE 1931 standard observer and illuminant D65.

The raw camera RGB of each patch in the color chart was obtained, and 50 × 50 pixels from the raw Bayer-patterned response of the central color patches of the image without postprocessing were extracted and demosaiced by the DcRaw program. Then, the transform matrix between the camera response and spectral reflectance was calculated from the training set and evaluated on the test set. To test the model more robustly and fairly, a ten-fold cross-validation approach was used ten times to evaluate the proposed method. All the patches were divided into ten groups randomly, and for each group, 9/10 of the patches were assigned as the training set, and 1/10 of the patches were assigned as the test set. Both spectral differences and color differences between the model-predicted results from the camera and the measurement results from the spectrophotometer were calculated to represent the performance of the predictive accuracy for the spectral reflectance estimation. That is, root-means-square error (*RMS*) was used as spectral metrics [15], with CIEDE2000 (color difference) under the D65 CIE standard illuminant as the colorimetric metric. The *RMS* and CIEDE2000 are positive values, with 0 corresponding to a perfect estimation. These metrics are given by the following equations:

(21) $$RMSE=\sqrt{\frac{1}{n}\sum_{i=1}^{n}{\left[r_{i}-\tilde{r_{i}}\right]}^{2}}$$

where $\tilde{r_{i}}$ denotes the reconstructed spectral reflectance of *i*th patches, $r_{i}$ denotes the measured reference of *i*th patches, and *n* denotes the number of full samples. *n* denotes the wavelength sampling number of the spectral reflectance over the visible spectrum.

### 3.2. The Influence of Feature Selection

Another common problem that was raised in the introduction section concerns the feature number of the expanded polynomial. For all the existing methods, the performance is calculated by the first-order, second-order, and third-order, rather than by the most important selected features. As noted in Section 2.3, the feature number of the camera response is crucial for spectral estimation.

**Figure 4:**
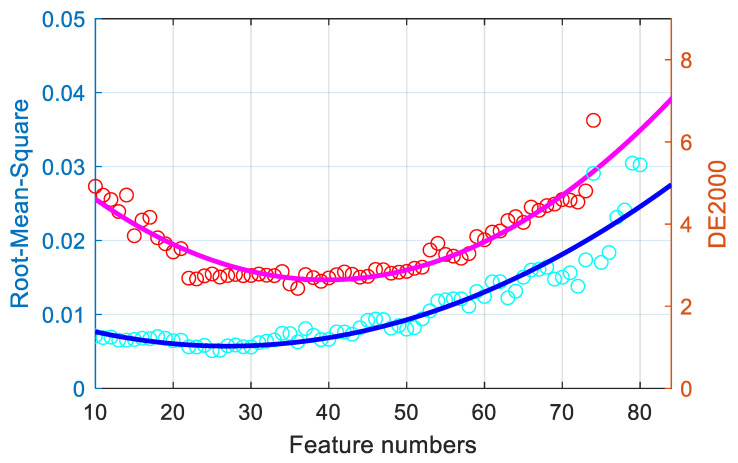
Relationship of the feature numbers with the spectral and color performance.

Neighborhood component analysis feature selection is performed to optimize the feature selection for the proposed imaging system. Here, the MATLAB function fsrnca, in the Machine Learning Toolbox, was used. However, as explained by Urban et al., too many items can cause overfitting problems [22]. By selecting weighted features, the colorimetric and spectral metric precision will be determined as the feature number is changed. In this study, using 380 color samples from the SG140 chart and DC240 chart, a regularization model with third-order polynomial expansion was used to calculate the different selected features in terms of both the spectral reflectance RMS and the mean CIEDE2000 color difference. Figure 4 reveals the relationship between the mean spectral reflectance RMS, the mean CIEDE2000 color difference, and the feature numbers within the expanded polynomial feature range. The performance of colorimetric and spectral metrics initially decreased with an increase in the feature number, and then their performance increased after an optimal value of approximately 30 features was reached. Thus, it should be more meaningful to understand the importance of the features and train a model using the only the selected features.

**Figure 5:**
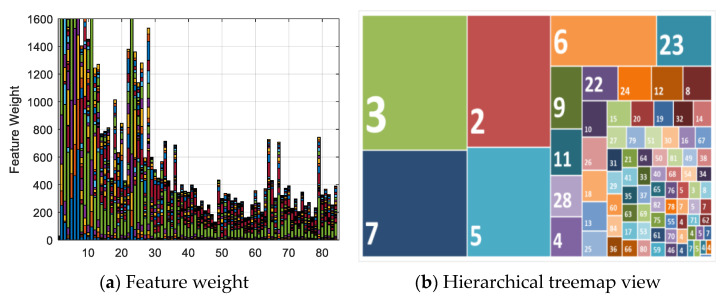
Feature selection among 84 items. (**a**) Feature weight of 84 extended feature items. (**b**) Hierarchical treemap view of feature weights.

Figure 5a illustrates the feature selection weights of 84 extended third-order polynomial feature items from six-channel camera responses using neighborhood component analysis (NCA). Over half of the feature weights are less than 400. Calculating the loss using the test set as a measure of the performance, the weights of the irrelevant features should be close to zero, and the performance of feature selection using five-fold cross-validation should improve. Figure 5b shows a hierarchical treemap view of the feature weights and shows the obvious patterns, including which items are most important for spectral estimation. The relationship between each feature is shown by color and proximity. The 30 most relevant features were identified and are summarized in Table 1. There are 6 first-order terms, 17 s-order terms, and 7 third-order terms that are most important for spectral estimation. This means that not all terms are necessary for spectral estimation; part of the second-order and third polynomial expansion are negative, irrelevant, and decrease the estimation performance. In addition, it should be noted that the selection of features might depend on the camera, illumination, and training dataset.

Based on the proposed multispectral camera system, the performance of the spectral reflectance estimation with different expansions (including the linear expansion, the second-order polynomial expansion, the third-order polynomial expansion, and finally, the third-order polynomial expansion with the proposed 30 features) was evaluated in terms of both the colorimetric error and spectral difference. All results are given in Table 2: compare the evaluation results of different polynomial expansion methods, it can be seen that the best performance, i.e., the smallest average color difference between the predicted and measured spectra, is achieved by selecting 30 feature items from the third-order polynomial expansion; it has the smallest value in root-means-square error (RMS), CIEDE2000 color difference under D65 illumination, lowest standard deviation (SD), and largest value in Student’s t variance (t-stat). Student’s t variance is used in the testing the variance for Student’s t distribution. The higher the t-stat value, the greater the confidence we have in the coefficient as a predictor. It is not safe to conclude that more feature numbers are needed to obtain more accurate results. Excessive training numbers would cause overfitting and thus reduce accuracy.

**Figure 6:**
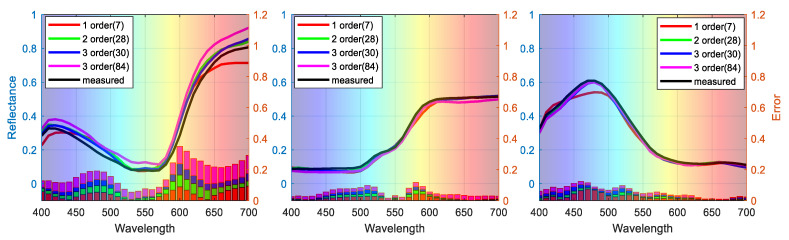
Representative samples of reconstructed spectra with different polynomial expansions.

To visualize the performance of different signal expansion methods, the estimated spectra of three randomly selected samples (No. 54, No. 180, and No. 288) are plotted in Figure 6, where the left axis shows the estimated spectra of samples with different polynomial expansions and the right axis shows the ΔR error between estimated and measured spectra. Our feature selection method outperforms the traditional expansion methods in terms of both spectral and colorimetric accuracy.

### 3.3. The Influence of the Regression Model on the Proposed Method

The proposed method was implemented and compared with currently existing methods. These included the regularized least-squares (RLS) [34], Tik regularized least-squares [36], Wiener method [5], ordinary least-squares method (OLS) [10], principal component analysis (PCA) [7], and partial least-squares method (PLS) [33]. All the existing methods were implemented in their optimal conditions, while six principal components were used conventionally for the PCA- and PLS-based methods, as the first six (instead of the total 84) components explain over 95% of the total variance.

Table 3 and Table 4 compare the evaluation results of the spectral metric in RMS and CIEDE2000 color difference between 84 items (third-order polynomial expansion) and 30 items (feature-selected). They show that our proposed method, the iteratively reweighted regularization model (IRWR), improves the precision of spectral reconstruction in color and spectral using feature selection, while the traditional methods exhibit a large discrepancy without considering the overfitting effect caused by an excessive number of features. Especially for the feature-selected model (30 items), IRWR has the smallest value in RMSE (mean value of 2.14%, maximum value of 9.35%, standard deviation (SD) of 1.77%) and smallest CIEDE2000 color difference (mean value is 1.79, the maximum value is 7.31, standard deviation (SD) is 1.39) compared to other methods. Furthermore, the predictive error of the feature-selected method is reduced by approximately 0.19 for the CIEDE2000 color difference compared to the unselected 84 items. Compared with the fully expanded items, our feature-selected model has the lowest standard deviation (SD), which indicates that the spectral estimation errors tend to be close to the mean value and the model has strong performance.

**Figure 7:**
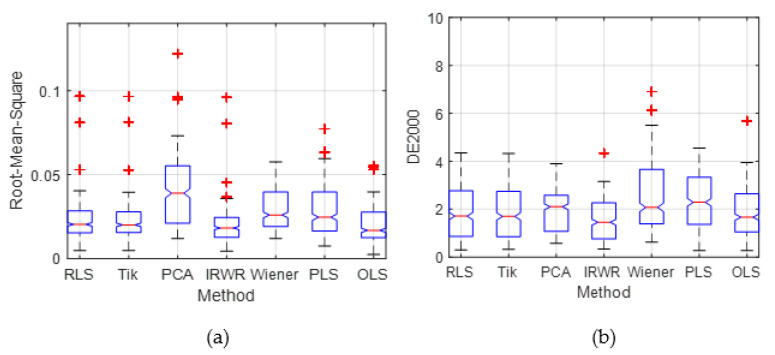
The relevant summary statistics of the proposed method and the existing methods, the outliers are plotted individually using the ‘+’ symbol. (**a**) Boxplot distributions of the RMS. (**b**) Boxplot distributions of the CIEDE2000 color difference.

To illustrate both the overall predictive accuracy and the error distribution of all methods, Figure 7 is plotted using boxplots to represent the distribution of the color difference and spectral RMS error compared with other estimation methods. The boxplot distribution of the estimation result of the proposed method is more compact than that of the existing methods, of which most of the CIEDE2000 and spectral RMS are less than those of the other traditional methods. The top of the blue rectangle indicates the upper quartile, a horizontal red line near the middle of the rectangle indicates the median, and the bottom of the blue rectangle indicates the lower quartile. The topmost outlier is represented by a vertical line that extends from the top of the rectangle to indicate the maximum value, and the bottom end outlier is represented by the vertical line that extends from the bottom of the rectangle to indicate the minimum value. The outliers (red cross) that are above or below the box body indicate that their values are greater than the upper quartile plus 1.5 times the interquartile range or less than the lower quartile minus 1.5 times the interquartile range. In addition, the boxplot of the overall error distribution for the ten-fold validation in Figure 7 shows that there are more small error samples estimated by the proposed method, which is more intuitive to prove the superiority of the proposed method.

**Figure 8:**
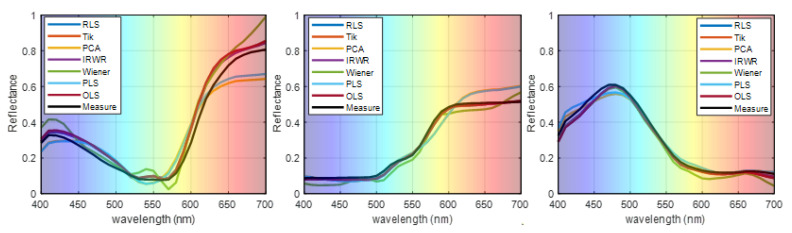
Representative samples of reconstructed spectra of the proposed method and the traditional methods.

The reconstruction of the spectral reflectance of three randomly selected samples (No. 54, No. 180, and No. 288) compared with those of the existing methods is shown in Figure 8, where the black line is the measured spectral reflectance. The reflectance reconstructed by the proposed method is found to be more accurate than those of the traditional methods. The experimental results using the simulated camera may, to some extent, prove the superiority of the proposed method.

### 3.4. Methods Implementation and Comparison for Illuminant Metamerism

To evaluate whether the RGB camera under two illuminations can give a better spectral estimation than that of the camera under one illumination, our proposed model with the third polynomial regression was also investigated under each illumination (3500 K or 6500 K) separately. The performance of the spectral estimation under 3500 K, under 6500 K, nonfeature selection (84 items), and feature selection (30 items) is listed in Table 5 in terms of the mean error, maximum and minimum error, standard deviation (SD), and Student’s t mean and variance (t-stat) of the color differences under these five test conditions. All the color difference data were calculated according to CIE Special Metamerism Index: Change in Illuminant (CIE 015.2-1986), and the data listed in the table were CIE ΔE*76 color difference between estimated and measured spectra.

**Figure 9:**
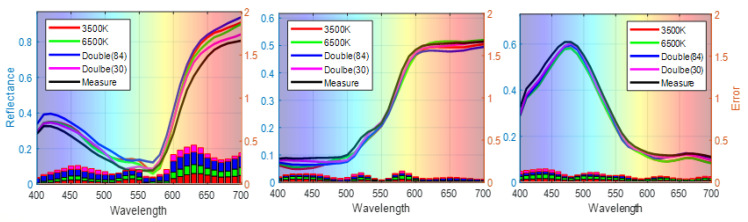
Representative samples of the reconstructed spectra in various conditions.

The results indicate that the three-channel signals of 3500 K and 6500 K with traditional quadratic expansions have high chromatic accuracy at the approximate color temperatures. The performance of the six channels under two illumination conditions is improved. It can be seen that the best performance, i.e., the smallest average color difference of color difference between the predicted and measured spectra, is achieved by our proposed method, which uses 30 items from the third-order polynomial expansion variables and six channels under both 3500 K and 6500 K illumination. The average color differences range from 2.14 (Illuminant D65) to 2.40 ΔE*ab units (Illuminant D50), with an overall mean of 2.23 ΔE*ab units, a maximum value of 5.41, and a standard deviation (SD) of 1.19. Additionally, the reconstructed spectral reflectance of the three randomly selected samples No. 54, No. 180, and No. 288) with single illumination (3500 K, 6500 K), double illumination, and feature-selected double illumination is shown in Figure 9. Our method achieves improved estimation accuracy by selecting features from polynomial expansions of camera response under two illumination conditions.

### 3.5. Discussion

In this study, we developed a cross-polarized multispectral camera system by applying two commonly used illuminants and propose a new method to improve the accuracy of the estimation of the spectral data from the raw camera responses. The results illustrate that the performance of reflectance estimation from the camera images can be significantly improved when two broadband illuminations are used. This implies that multi-illumination with consumer-grade cameras can be an effective approach to construct a multispectral camera system. The factors that have contributed to the improvement in estimation accuracy are as follows.

(1) We found that the feature selection of the expanded camera response influences the estimation performance. We have shown that a small number of features can provide better performance than the full selection of features of the camera response expansion. The selection of features might depend on the camera, illumination, and training dataset. Further investigation of factors affecting the feature selection was conducted and reported in this paper.

(2) As outliers present in the training data could lead to poor estimates, the iteratively reweighted regulated model was proposed. An analysis of residuals was necessary to work around this by assigning weights to the training data. The weighting is done automatically and iteratively; weights are recomputed iteratively so that the points farther from the model predictions in the previous iteration are given a lower weight. Then, the influence of outliers is downweighted. The result supported our previous research.

(3) The most significant performance improvement was achieved by mapping the six-channel signals under two illumination conditions into the spectral reflectance, which minimized the degree of metamerism significantly compared to the three-channel mapping. As shown in Table 5, for each of the illumination conditions tested, the best performance, i.e., the smallest mean color difference between the predicted and the measured spectral data, was achieved by using six channels under two illumination conditions, where the mean color differences ranged from 2.14 (Illuminant D65) to 2.4 ΔE*ab units (Illuminant D50), with an overall average of 2.23 units.

## 4. Conclusions

This paper proposes a method for spectral estimation to calculate the spectral data from raw camera responses by feature-selected expansion items under two illumination conditions using an iteratively reweighted regulated model. The performance of the proposed method is evaluated using ColorChecker charts. The results show that our proposed method achieves good accuracy in terms of both spectral and colorimetric estimation. The factors that contributed to the proposed method are discussed in detail; downweighting the influence of the outliers in the training set and selecting some of the most important features obviously improves the performance of spectral estimation. However, there are still problems to be solved in the future. For example, our method can be slightly worse than some local weighted regression methods, although it is less computationally expensive than the traditional methods. The tradeoff between accuracy and computational complexity is the most common shortcoming for nearly all adaptive methods. The proposed method has potential applications for spectral reflectance measurement in many fields including textiles, printing, and cultural heritage.

## Figures and Tables

**Table 1:** Selected terms of the polynomial regression.

Order	Polynomial Regression
1st-order (6)	$R_{1},G_{1},B_{1},R_{2},G_{2},B_{2}$
2nd-order (18)	$\begin{array}{l} R_{1}^{2},G_{1}^{2},R_{2}^{2},G_{2}^{2},B_{2}^{2},R_{1}B_{2},R_{1}G_{2},R_{1}R_{2},R_{1}B_{1},G_{1}B_{2},\\ G_{1}G_{2},G_{1}R_{2},B_{1}R_{2},B_{1}B_{2},B_{1}G_{2},R_{2}G_{2},B_{2}G_{2},B_{2}R_{2} \end{array}$
3rd-order (6)	$B_{2}^{3},B_{2}^{2}R_{2},B_{2}^{2}R_{1},B_{2}R_{2}^{2},B_{2}R_{1}R_{2},B_{2}R_{1}^{2}$

**Table 2:** Performance of reflectance estimation using two illuminants with different polynomial expansions.

	RMS						CIE DE00			
	Mean	Min	Max	SD	T-Stat	Mean	Min	Max	SD	T-Stat
1st-order (7)	2.85%	0.48%	39.2%	0.03	21.6	2.09	0.08	32.1	2.0	20.0
2nd-order (28)	2.24%	0.25%	69.3%	0.04	11.7	2.11	0.17	141.9	7.3	5.6
3rd-order (84)	2.18%	0.23%	107.5%	0.06	7.4	1.97	0.12	131.1	6.9	5.6
Selected (30)	2.14%	0.18%	34.4%	0.02	19.3	1.79	0.11	20.2	1.5	22.9

**Table 3:** The comparison of estimation accuracy in terms of RMS using ten-fold cross-validation.

Model	84 Items (3rd-Order Polynomial Expansion)				30 Items (Feature-Selected)			
	Mean (%)	Max (%)	Min (%)	SD (%)	Mean (%)	Max (%)	Min (%)	SD (%)
RLS [34]	2.84	18.37	0.37	3.39	2.88	28.06	0.38	4.88
Tik [36]	2.91	30.70	0.37	5.26	2.81	25.66	0.38	4.49
PCA [7]	3.77	11.45	0.84	2.48	3.78	12.09	0.96	2.53
Wiener [5]	4.88	20.13	1.16	3.89	3.54	20.96	0.85	3.53
PLS [33]	3.81	11.25	0.79	2.49	3.68	10.91	0.76	2.39
OLS [10]	2.99	24.63	0.35	4.27	2.30	17.39	0.36	2.99
IRWR	2.34	16.24	0.36	2.89	2.14	9.35	0.35	1.77

**Table 4:** Comparison of estimation accuracy in terms of CIEDE2000 using ten-fold cross-validation.

Model	84 Items (3rd-Order Polynomial Expansion)				30 Items (Feature-Selected)			
	Mean (%)	Max (%)	Min (%)	SD (%)	Mean (%)	Max (%)	Min (%)	SD (%)
RLS [34]	2.35	15.32	0.27	2.70	2.30	19.06	0.39	3.30
Tik [36]	2.31	21.73	0.34	3.69	2.32	19.99	0.38	3.42
PCA [7]	2.16	6.16	0.41	1.23	2.17	6.05	0.39	1.22
Wiener [5]	4.85	20.91	0.69	4.64	2.95	19.85	0.48	3.61
PLS [33]	2.28	6.65	0.41	1.36	2.26	6.54	0.41	1.34
OLS [10]	2.65	24.73	0.27	4.19	2.07	20.26	0.26	3.44
IRWR	1.98	19.5	0.23	3.37	1.79	7.31	0.33	1.39

**Table 5:** Metamerism performance of four methods under different illuminations.

Illumination Temperature (Items)		3500 K (10)	6500 K (10)	3500 K + 6500 K (30)	3500 K + 6500 K (84)
A	Mean	2.65	2.85	2.39	2.18
	Max	7.09	7.67	8.12	5.35
	Min	0.27	0.22	0.38	0.30
	SD	1.53	1.70	1.56	1.10
	t-stat	10.58	10.23	9.30	12.01
F2	Mean	2.94	2.58	2.47	2.31
	Max	9.72	7.12	8.55	5.35
	Min	0.32	0.26	0.29	0.18
	SD	2.08	1.66	1.71	1.25
	t-stat	8.60	9.42	8.80	11.22
TL84	Mean	2.65	2.49	2.35	2.15
	Max	7.64	6.03	8.56	5.31
	Min	0.27	0.21	0.31	0.19
	SD	1.65	1.49	1.62	1.16
	t-stat	9.77	10.12	8.79	11.24
D50	Mean	2.92	2.81	2.62	2.40
	Max	7.93	7.04	9.69	5.72
	Min	0.25	0.24	0.56	0.32
	SD	1.85	1.66	1.80	1.23
	t-stat	9.60	10.26	8.85	11.83
D65	Mean	2.74	2.40	2.34	2.14
	Max	7.87	5.77	8.77	5.30
	Min	0.26	0.21	0.28	0.19
	SD	1.77	1.46	1.65	1.19
	t-stat	9.42	9.97	8.62	10.99
Mean (above illumination)	Mean	2.78	2.62	2.43	2.23
	Max	8.05	6.73	8.74	5.41
	Min	0.28	0.23	0.36	0.24
	SD	1.77	1.60	1.67	1.19
	t-stat	9.59	10.00	8.87	11.46

## Data Availability

Data available in a publicly accessible repository. The data presented in this study are openly available in [https://zenodo.org/record/5730472#.YaE3PhrP23A] (accessed on 24 November 2021) at [DOI 10.5281/zenodo.5730471].
